# Neurotoxic Effects of Tramadol Administration on the Cerebellar and Cerebral Cortices of Adult Male Wistar Rats

**DOI:** 10.7759/cureus.86904

**Published:** 2025-06-28

**Authors:** Aliyu O Olaniyi, Abdullateef O Yusuf, Kingsley U Ogbe, Musa O Iliyasu, Suleiman M Eze, Abdulqudus O Omirude, Madhumati Mandal

**Affiliations:** 1 Geriatrics, Stepping Hill Hospital, Stockport NHS Foundation Trust, Manchester, GBR; 2 Anatomy, Faculty of Basic Medical Sciences, Prince Abubakar Audu University, Anyigba, NGA; 3 Animal and Environmental Biology, Prince Abubakar Audu University, Anyigba, NGA; 4 Anatomy, Al-Hikmah University, Ilorin, NGA; 5 Biomedical Sciences, National Horizons Centre, Teesside University, Middlesbrough, GBR; 6 Anatomy/Neuroscience, Ahmadu Bello University, Zaria, NGA

**Keywords:** cerebellar, cerebral, neurodegeneration, oxidative biomarkers, tramadol

## Abstract

Background

Tramadol, a widely prescribed synthetic opioid, is commonly used for managing moderate to severe pain due to its dual mechanism of action as a μ-opioid receptor agonist and norepinephrine and serotonin reuptake inhibitor.

Aim

To evaluate the neurotoxic effects of tramadol administration on the cerebellar and cerebral cortices of adult male Wistar rats, focusing on behavioural changes, oxidative stress markers, and histopathological alterations.

Materials and methods

Twenty adult male Wistar rats were divided into four groups of five rats each: a control group and three treatment groups receiving 25 mg/kg, 50 mg/kg, and 75 mg/kg of tramadol orally, respectively, for 21 days. Physical behaviour was monitored. Oxidative stress markers were assessed. Histological examination of the cerebellum and cerebrum was also carried out.

Results

The tramadol-treated groups exhibited increased aggression and significant weight loss, particularly at higher doses. Oxidative stress markers showed no changes in the antioxidant enzymes assayed with the exception of catalase. Histopathological examination of the cerebellum showed intact cerebellar layers at the cortex with normal cellular structure and orientations for the control and treated group, while that of the cerebrum revealed mild morphological alterations in the cerebrum of tramadol-treated rats compared to the control group.

Conclusion

These findings suggest that tramadol exposure can lead to neurotoxic effects in the cerebrum of rats. The observed morphological and histological changes may contribute to the development of long-term adverse effects associated with tramadol use, such as cognitive impairment and mood disorders.

## Introduction

Tramadol is commonly prescribed for chronic pain conditions due to its efficacy and lower potential for addiction compared to other opioids [1]. However, concerns about its neurotoxic effects have led to increased interest in studying its impact on the central nervous system, including the cerebellum and cerebrum.

Tramadol can lead to physical dependence and withdrawal symptoms, which include neurological manifestations such as anxiety, tremors, and insomnia. These withdrawal symptoms can further complicate cerebral health by contributing to psychological stress and potential relapse of other neurological conditions [2]. There is some evidence suggesting that tramadol may have neurotoxic effects, particularly with prolonged use or overdose [3].

The cerebellum is a critical part of the brain involved in motor control, coordination, balance, and cognitive functions. It receives sensory input from the spinal cord and other parts of the brain and integrates this information to fine-tune motor activity [4]. Any damage or alteration to cerebellar function can result in ataxia, dysmetria, and other motor deficits.

The cerebrum is part of the central nervous system situated in the cranial cavity and connected to the cerebellum through the brainstem [5]. It is the largest part of the brain that is responsible for processing sensory information, controlling movement, and managing higher-level cognitive functions [6]. Despite its analgesic benefits, there is growing concern about its potential neurotoxic effects, particularly on the cerebrum. This concern is pertinent given the evidence suggesting that tramadol can induce seizures, contribute to serotonin syndrome, and impair cognitive functions in humans [2].

Previous research has shown that tramadol can alter brain function and structure, particularly in relation to pain processing and motor control [7]. Long-term tramadol use has been linked to changes in brain chemistry and function, including adaptation in opioid receptor expression and signaling pathways [8]. Studies using neuroimaging techniques like functional magnetic resonance imaging (FMRI) have also demonstrated that tramadol can modulate brain activity in response to pain stimulation [9]. Additionally, tramadol has been found to affect various brain regions, including the limbic system, brainstem, and cerebral cortex leading to changes in pain perception, mood regulation, and cognitive function [10]. This study aims to evaluate the effects of tramadol on the cerebellar and cerebral cortices of adult male Wistar rats, focusing on behavioural changes, haematological indices, oxidative stress markers, and histopathological alterations.

## Materials and methods

Experimental animals

Twenty (20) adult male Wistar rats were used for this study. The rats were kept in adequately ventilated cages, provided with essential feeds and water, in a regular laboratory setting. A period of two weeks was allowed for the animals to acclimatize to the environment.

Drugs

The following drugs were used in the study:

i. Tramadol tablets

ii. Chloroform (anesthesia)

Other materials

Additional materials employed in this study comprised a weighing scale, cages, sawdust for bedding, water bottles, feeding bowl, distilled water, dissection tools, a light microscope, syringes of varying volumes (20 ml, 5 ml, and 1 ml), protective gloves, cotton wool, alcohol, glass slides, tissues, cover slips, containers for tissue fixation, fixatives and stains, a spectrophotometer, and 0.1M Phosphate Buffer Saline (PBS), among others.

Experimental design

A total of twenty (20) adult male Wistar rats were used for this study, which were weighed and then randomly allocated into four separate groups in their home cages, each experimental group containing four rats. The administration was based on the rats' respective body weight, and the administration of tramadol was via the oral route.

The experimental design and grouping are presented in Table 1.

**Table 1:** Treatment of Experimental Rats n=5; all administration was via the oral route. Administration lasted for three weeks (21 days).

Groups	Treatment
I (Control)	1 mg/kg H2O
II (Tramadol Low dose)	25 mg/kg Tramadol
III (Tramadol Medium dose)	50 mg/kg Tramadol
IV (Tramadol High dose)	75 mg/kg Tramadol

The experimental procedures were carried out in the histology laboratory, Department of Human Anatomy, College of Health Sciences, Prince Abubakar Audu University, Anyigba, unless specified otherwise.

Tissue collection

Euthanasia

Euthanasia of the rats was conducted using chloroform inhalation in accordance with ethical guidelines. Chloroform, a volatile liquid, was administered using a suitable euthanasia chamber specifically designed for small animals. The rats were exposed to a controlled concentration of chloroform vapor until they became deeply anesthetized and subsequently subjected to euthanasia.

Tissue Preservation

Brain tissues of the experimental Wistar rats were fixed in neutral-buffered formalin, ensuring complete coverage of the tissues.

Histological studies

Tissue processing was carried out using a standard H&E staining protocol for general tissue morphology. Microscopic examination of stained sections and capturing representative photomicrographs was done with the aid of a light microscope.

Biochemical studies

Oxidative Biomarkers

Measurement of oxidative stress biomarkers was conducted as part of the study. Serum obtained from the animals was used to assay antioxidant level of Malondialdehyde (MDA), was assessed alongside the activity of antioxidant enzymes such as superoxide dismutase (SOD) and glutathione (GSH).

Measurement of Catalase Activity

Catalase activity was determined using the method described by Sinha [11]. The procedure involved the use of 5% potassium heptaoxochromate (VI) K2Cr2O7, which was mixed with glacial acetic acid in a ratio 1:3. This mixture was stored in a brown bottle at room temperature. About 0.9 ml of distilled water was then added to 0.1 ml of the sera, and this was mixed thoroughly. A 2.5 ml of phosphate buffer was placed in a conical flask to which 0.5 ml of sera was added, and 2.0 ml of H2O2 was added as well. The reaction mixture was thoroughly mixed. A stopped watch was used to monitor the reaction, and the reaction was stopped after every 60 seconds for 3 minutes using a dichromate/acetic acid solution. This mixture was heated in a water bath for 10 minutes at 80°C. Absorbance was read at 570 nm. A standard curve was obtained using the absorbance obtained at various H2O2.

Calculations: The quantity of H2O2 consumed was obtained from the graph of the catalase standard curve; this determined the catalase activity.

Measurement of Superoxide Dismutase Activity

Superoxide dismutase (SOD) activity was determined by a method described by Fridovich [12]. In this method, 0.1 ml of sera was diluted in 0.9 ml of distilled water to make 1:10 dilution of sera. An aliquot mixture of 0.20 ml of the diluted sera was added to 2.5 ml of 0.05 M Carbonate buffer. The reaction commenced with the addition of 0.3 ml of 0.3 mM adrenaline. The reference solution contains 2.5 ml of 0.05 M Carbonate buffer, 0.3 ml of 0.3 mM adrenaline and 0.20 ml of distilled water. The absorbance was read over 30 s up to 150 s at 480 nm.

Calculations: Increase in absorbance per minute = (A5 - A1)/2.5

% inhibition = 100 - Increase in absorbance for substrate x 100 Increase in absorbance of blank

1 unit of SOD activity is the quantity of SOD necessary to elicit 50% inhibition of the oxidation of adrenaline to adrenochrome in 1 minute.

Assessment of Malondialdehyde (MDA)

MDA is an index of lipid peroxidation as evidenced by the formation of thiobarbituric acid reactive substances (TBARS) measured by the modified method of Niehaus and Samuelsson (1968) [13]. In this method, 150 µl of sera (O.25M sucrose solution) was treated with 2 ml of (1:1:1 ratio) TBA-TCA-HCL reagent (thiobarbituric acid 0.37%, 0.25N HCL and 15% TBA) and placed in a hot water bath for 1 hr at 90°C. The reaction mixture was cooled and centrifuged at 3000 rpm for 5 min at 4°C. Absorbance of the pink coloured supernatant (2.0 ml) was measured against a reference blank with a spectrophotometer at 535 nm.

Calculations: The MDA was calculated using the molar extinction coefficient of 1.56 x 10^5^ cm^-1^M^-1^

MDA conc. = absorbance/ 1.56 x 10^5^ cm^-1^M^-1^ x l

Measurement of Reduced Glutathione Concentration

Reduced glutathione (GSH) concentration was carried out according to the method of Ellman (1959) [14]. For the assay, 0.2M phosphate buffer: 8.40 g of NaH2PO4 and 9.94 g of Na2HPO4 were dissolved in distilled water and made up to 1000 ml mark of a volumetric flask; the buffer was adjusted to pH 8.

To 150 µl of serum (in PBS, pH 7.4), 1.5 ml of 10% trichloroacetic acid (TCA) was added. This mixture was centrifuged at 1500 g for 5 min. A 1 ml of the resulting supernatant was treated with 0.5 ml of Ellman’s reagent (19.8 mg of 5, 5’-dithiobis (nitrobenzoic acid) (DNTB) in 100 ml of 0.1% sodium nitrate) as well as 3 ml of phosphate buffer (0.2 M, pH 8). The absorbance was measured at 412 nm.

Statistical analysis

All the results were analyzed using GraphPad Prism version 9.3.1 (GraphPad Software, San Diego, CA, USA), and the results were expressed as Mean ± SEM. The statistical significance between means was analyzed using one-way analysis of variance (ANOVA) followed by the Tukey post hoc test. P-value < 0.05 was considered statistically significant.

## Results

Physical observations

There were no changes observed physically in the control group, which is group 1, after administration of distilled water; the activity of the animals was normal, and no reduction in their feeding habit within the group. However, progressive aggressive behavior was observed across the treatment groups (groups 2, 3 and 4) during the administration of tramadol. In addition, there was a decrease in the body weight of animals in these groups.

Oxidative biomarkers

Malondialdehyde (MDA) Concentration

**Figure 1:**
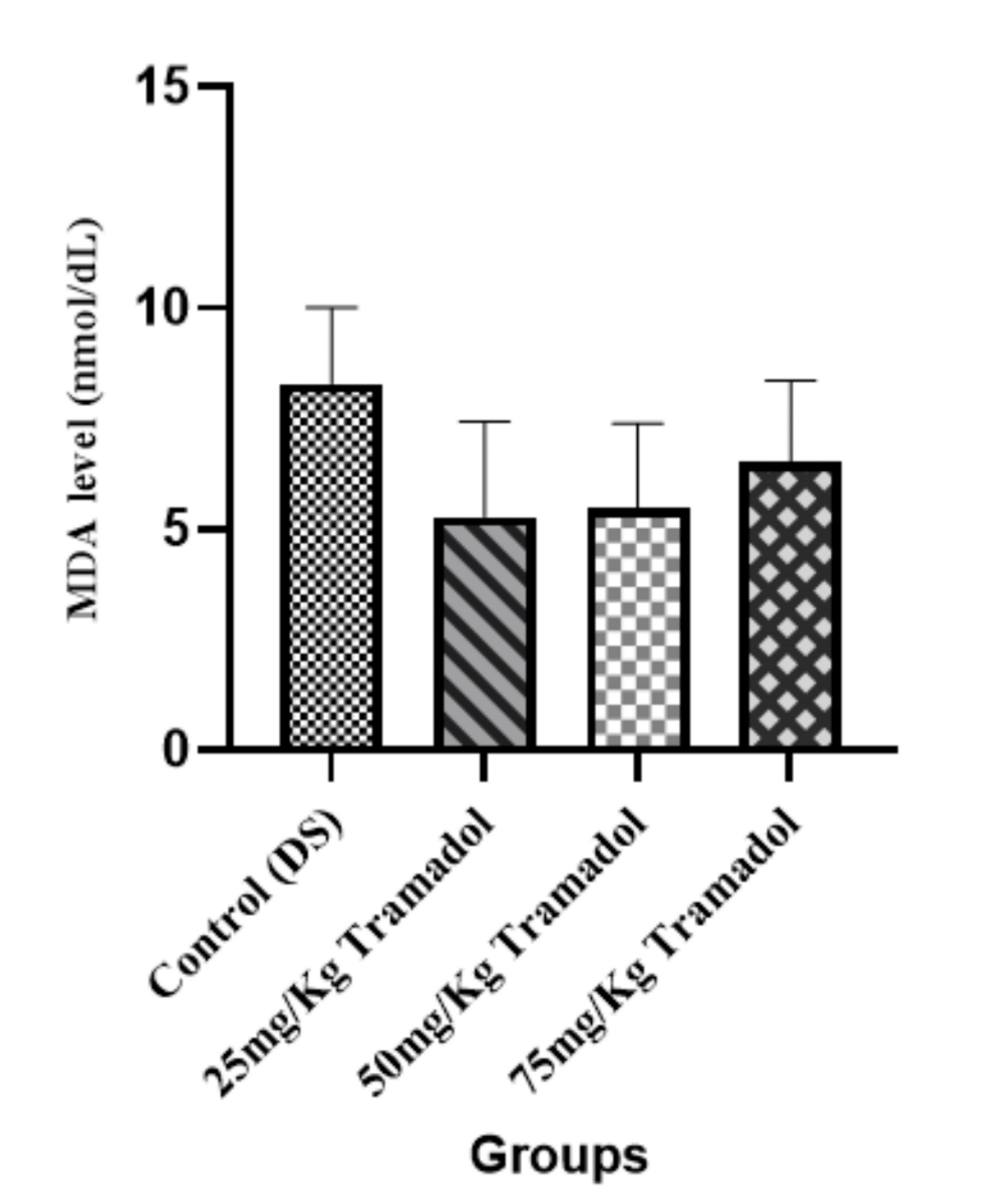
Malondialdehyde concentration for male adult Wistar rats following tramadol administration n=5, mean ± SEM, One way ANOVA, Tukey post hoc test; p> 0.05

Figure 1 indicates that MDA is a marker of lipid peroxidation, indicating oxidative stress levels. No significant difference (p > 0.05) in MDA levels was found between the control group and the tramadol-treated groups, suggesting that tramadol at the administered doses did not significantly increase lipid peroxidation in the experimental rats.

Catalase Activity

**Figure 2:**
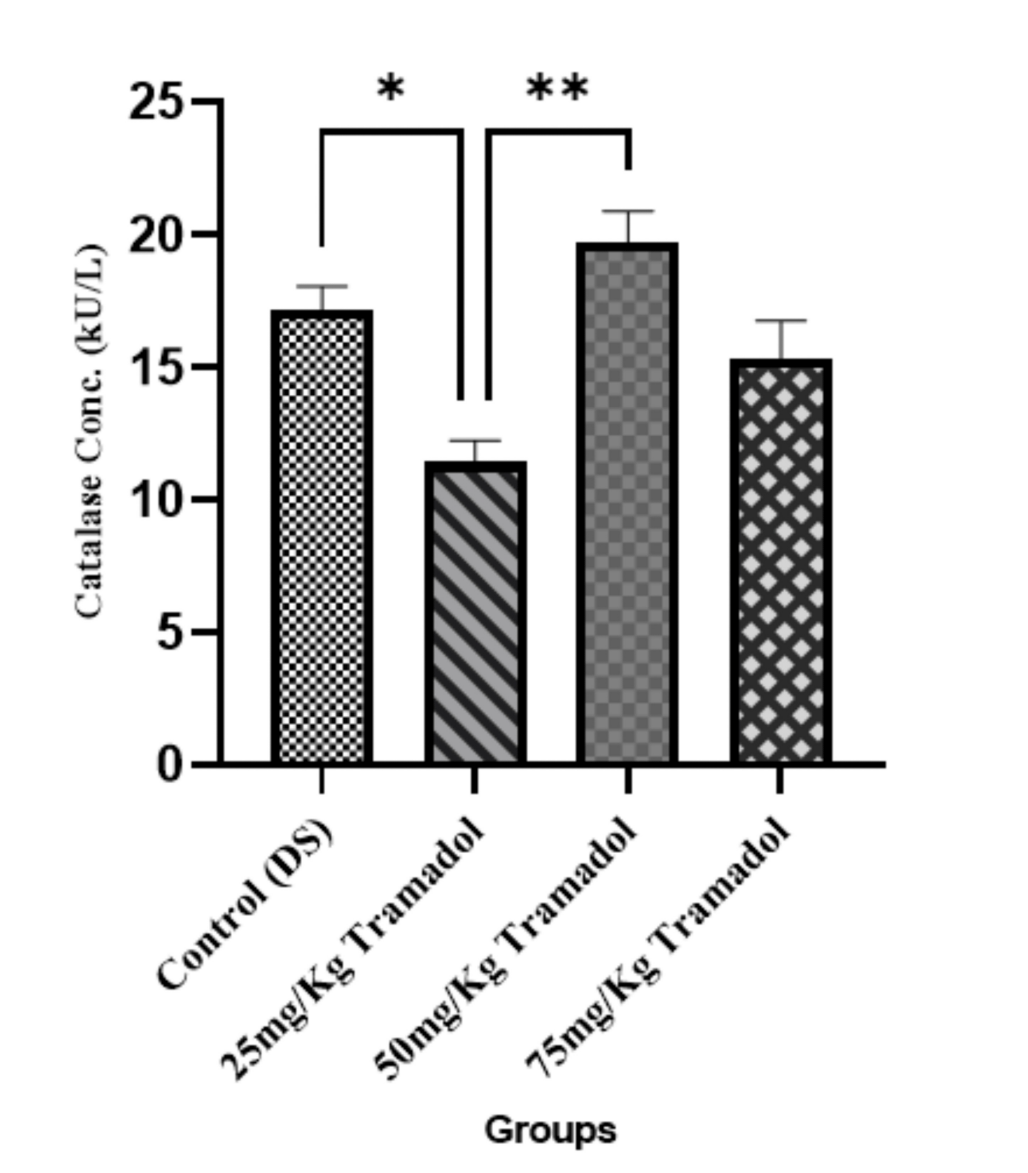
Catalase activity for male adult Wistar rats following tramadol administration n=5, mean ± SEM, One way ANOVA, Tukey post hoc test; *= p< 0.05, **= p< 0.01

Catalase is an enzyme that helps neutralize oxidative stress by breaking down hydrogen peroxide into water and oxygen. Figure 2 shows a significant increase in catalase activity as observed in the tramadol-treated groups compared to the control group (p < 0.05 and p < 0.01). This suggests that tramadol ingestion induced an oxidative stress response, leading to increased catalase activity as a protective mechanism.

Superoxide Dismutase (SOD) Activity

**Figure 3:**
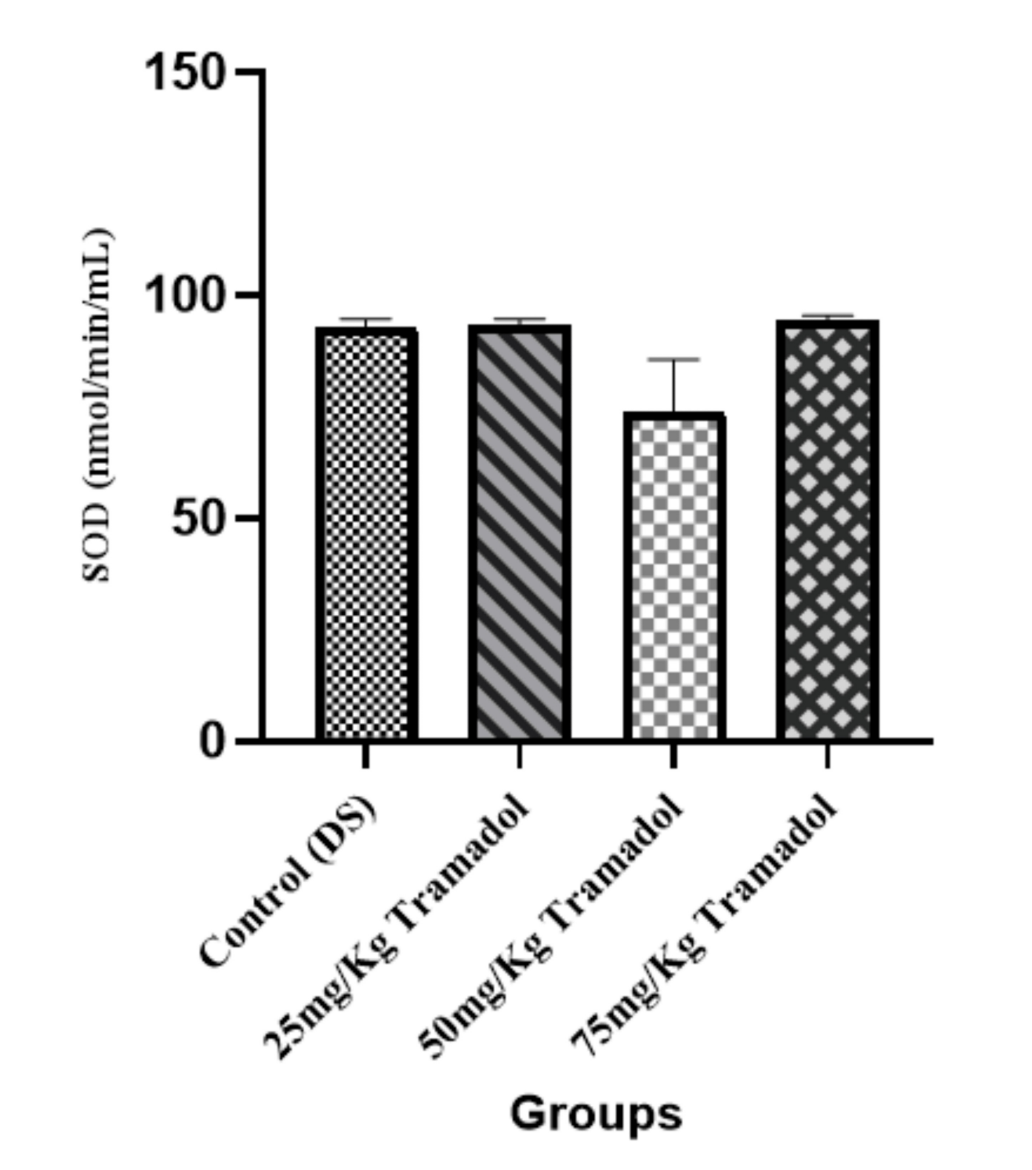
Superoxide dismutase activity for male adult Wistar rats following tramadol administration n=5, mean ± SEM, One way ANOVA, Tukey post hoc test; p> 0.05 SOD: Superoxide dismutase

Figure 3 shows the result of SOD activity. SOD is another critical antioxidant enzyme that helps mitigate oxidative stress by converting superoxide radicals into less harmful molecules. There was no significant change (p > 0.05) in SOD activity across the groups, indicating that tramadol did not significantly affect SOD activity in the experimental rats.

Reduced Glutathione (GSH) Concentration

**Figure 4:**
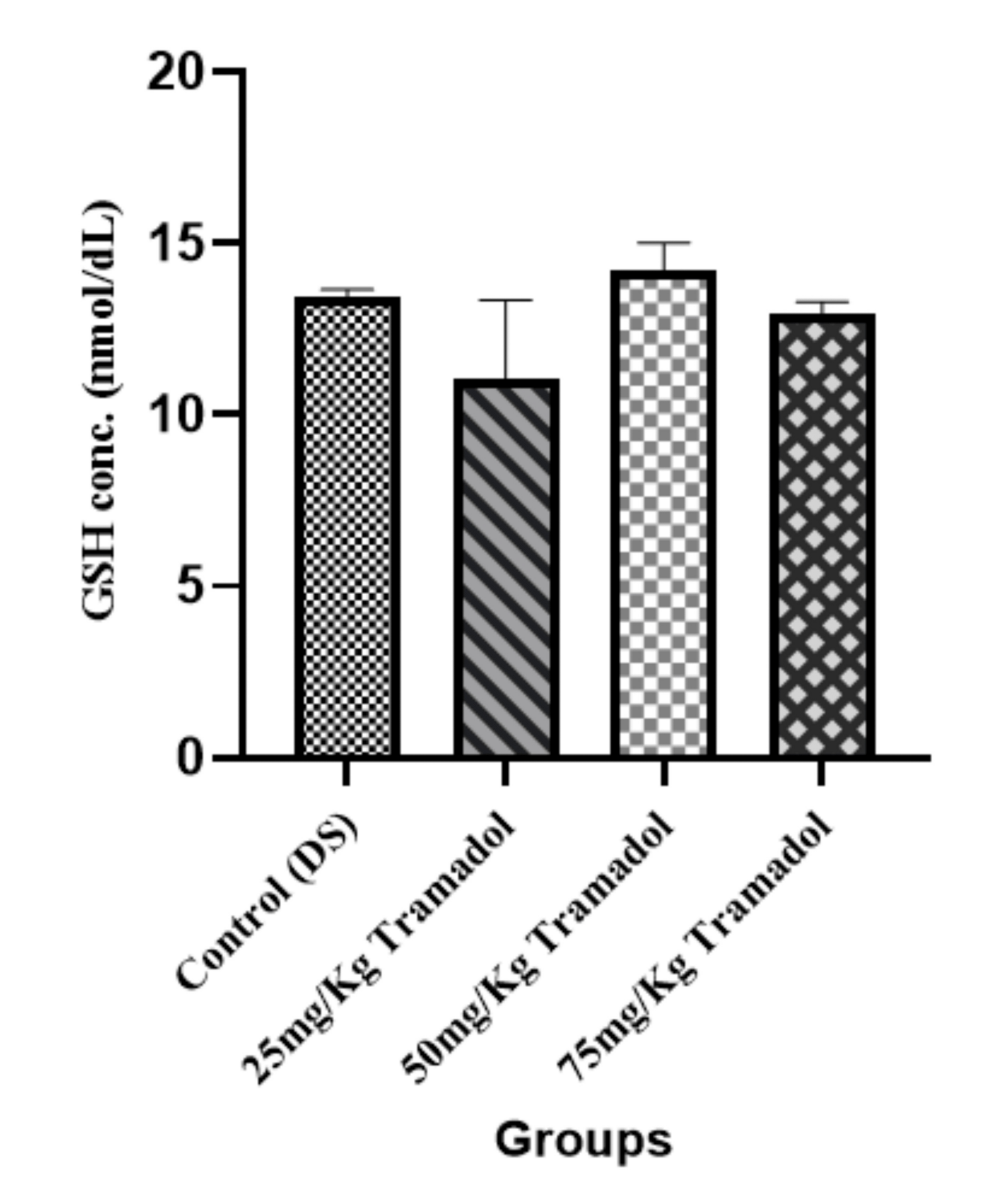
Reduced glutathione concentration for male adult Wistar rats following tramadol administration n=5, mean ± SEM, One way ANOVA, Tukey post hoc test; p> 0.05 GSH: Reduced glutathione

Figure 4 shows the result of GSH concentration. GSH is a vital intracellular antioxidant that protects cells from oxidative damage. No significant difference (p > 0.05) was noted in GSH levels between the groups, suggesting that tramadol ingestion did not significantly deplete GSH reserve in the experimental rats.

Histology of the cerebellum

Control Group (1 ml/kg H2O)

**Figure 5:**
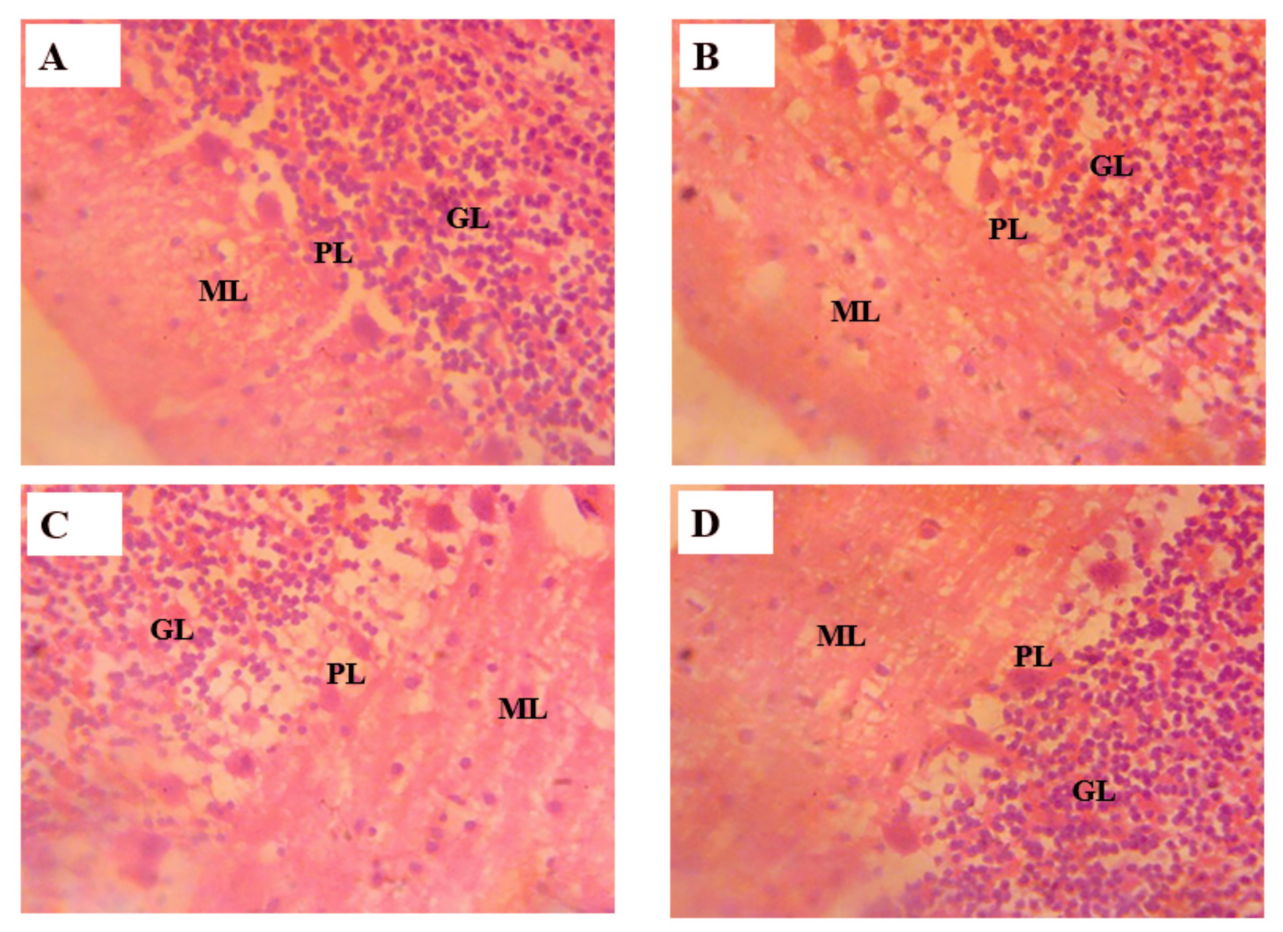
Photomicrograph of the cerebellar cortex of adult male Wistar rats following administration of tramadol. (A) Control group (distilled water), (B) treated group 2 (25 mg/kg of tramadol), (C) treated group 3 (50 mg/kg of tramadol), (D) treated group 4 (75 mg/kg of tramadol). ML: Molecular layer, PL: Purkinje cell layer, GL: Granule cell layer H&E stain x400

Figure 5A shows that the Purkinje cells in this group appear normal, with clearly defined cell bodies. No signs of degeneration or damage are observed, indicating that the cerebellar structure is intact and functioning properly. The molecular layer shows a uniform distribution of neuronal processes. The layer is well-preserved with no signs of cellular disruption. In the granule cell layer, the granule cells are densely packed and uniformly distributed, showing no signs of cell loss or damage. The overall structure of the cerebellum in the control group remains intact, reflecting normal cerebellar histoarchitecture.

Low Dose Tramadol (25 mg/kg)

Figure 5B, when compared to the control group, reveals that the Purkinje cells in this group appear normal, with clearly defined cell bodies. No signs of degeneration or damage are observed, indicating that the cerebellar structure is functioning properly. The molecular layer shows an even distribution of neuronal processes, including that of Purkinje cells. The layer is well-preserved with no signs of cellular disruption. In the granule cell layer, the granule cells are still densely packed and uniformly distributed, showing no signs of any cell loss or damage. The structures of the cerebellum in this group show no damage, indicating normal cerebellar function.

Medium Dose Tramadol (50 mg/kg)

Figure 5C, compared to the control group, shows that the Purkinje cells in this group show no abnormality, with clearly defined cell bodies. No signs of degeneration or damage are observed, indicating that the cerebellar structure is still functioning properly. The molecular layer shows an undisrupted distribution of neuronal processes, including that of Purkinje cells. The layer is preserved with no signs of cellular disruption. In the granule cell layer, the granule cells are densely packed and uniformly distributed, showing no signs of any cell loss or damage. The structures of the cerebellum in this group show no damage, indicating that cerebellar architecture is normal.

High Dose Tramadol (75 mg/kg)

In Figure 5D, compared to the control group, the Purkinje cells in this group begin to show mild changes. While the majority of cells still appear normal, some Purkinje cells exhibit slight shrinkage and reduced dendritic arborization. This indicates the beginning of potential neurotoxic effects due to high-dose of tramadol. The molecular layer in this group shows minor disruptions. The layer is still largely intact, but there are early signs of reduced synaptic interactions. In the granule cell layer, there is a slight reduction in granule cell density, but the changes are minimal. The granule cells continue to function, though there is a mild indication of stress or early signs of damage.

Histology of the cerebrum

**Figure 6:**
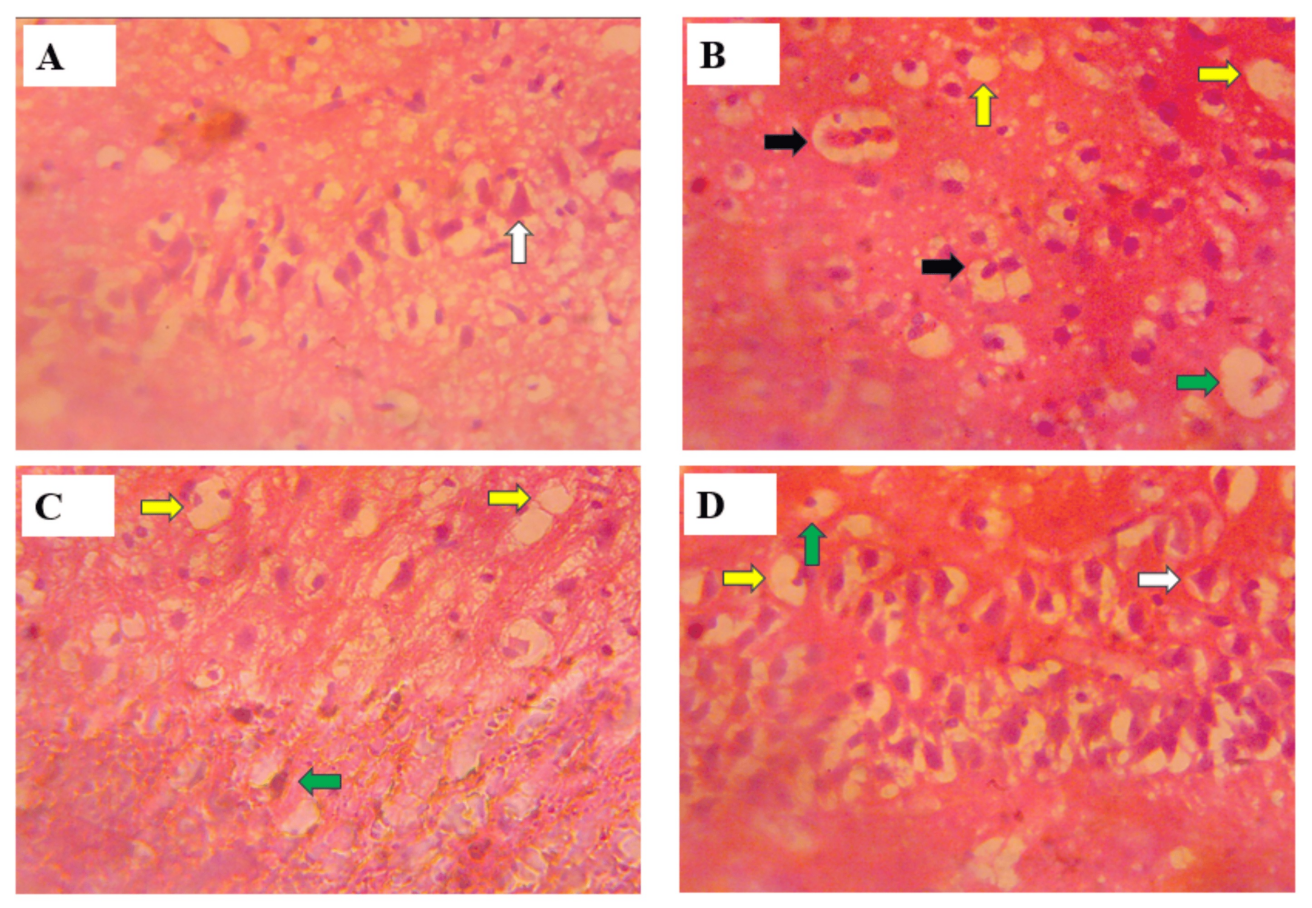
Photomicrograph of the cerebral cortex of adult male Wistar rats following tramadol administration (A) Control group (distilled water), (B) treated group 2 (25 mg/kg of tramadol), (C) treated group 3 (50 mg/kg of tramadol), (D) treated group 4 (75 mg/kg of tramadol). White arrow = normal Pyramidal cell, green arrow = pyknosis, yellow arrow = vacuolation, black arrow = karyorrhexis. H&E x400

The photomicrograph of the stained cerebral tissue revealed some neurodegenerative changes in the forms of necrosis, pyknosis, vacuolations and karyorrhexis among the treated groups. The control group, however, showed no histopathological changes. Figure 6A shows the intact or normal architecture of the cerebral cortex, with prominent pyramidal cells and less vacuolation. Figure 6B, which represents 25 mg/kg of tramadol administration, reveals a mild presence of pyknosis, vacuolations and necrotic nuclei when compared to the control group. Figure 6C shows an increased level of vacuolations and pyknosis in the cerebral cortex of treated rats (50 mg/kg of tramadol) when compared to group II. Figure 6D reveals similar histopathological changes to that of Figure 6C in the cerebral cortex of Wistar rats following tramadol treatment (75 mg/kg of tramadol).

## Discussion

Tramadol is a synthetic opioid commonly prescribed for the management of moderate to severe pain, primarily due to its dual mechanism of action as a weak μ-opioid receptor agonist and an inhibitor of norepinephrine and serotonin reuptake. While tramadol is considered safer than other opioids, particularly regarding its lower potential for addiction and respiratory depression, emerging evidence suggests that it may still pose significant neurotoxic risks, particularly with long-term use as a result of its abuse among youths. Previous studies have demonstrated that opioids can induce neurodegenerative changes in the brain, largely through mechanisms involving oxidative stress and apoptosis [7].

Physical observation

In this study, the physical observations revealed notable behavioural changes in the rats administered tramadol, especially in the medium and high-dose groups. The treated groups exhibited increased aggression and a significant decrease in body weight. These changes suggest that tramadol may induce stress responses and metabolic disruptions, consistent with findings from other studies where opioids caused similar behavioural alterations [15].

Oxidative biomarkers

Tissue damage mediated by oxidative stress is an established neuro-pathological mechanism [16]. The evaluation of oxidative biomarkers revealed a dose-dependent increase in malondialdehyde (MDA) levels, a marker of lipid peroxidation, alongside a decrease in the activity of antioxidant enzymes such as superoxide dismutase (SOD) and glutathione (GSH). These findings indicate that tramadol administration leads to significant oxidative stress in the Wistar rats, which is a critical factor in the observed neurodegenerative changes. Similar findings were reported by Barbosa et al. [17], who found that opioids induce elevated oxidative stress markers in the brain, leading to neuronal apoptosis and structural damage. The decrease in antioxidant enzyme activity observed in this study suggests that the cerebellum’s defense mechanisms against oxidative damage are compromised under tramadol exposure, thereby increasing its susceptibility to neurotoxicity.

Histology of the cerebellum

Photomicrograph of the cerebellum of the control group revealed distinct cellular outlines of molecular layer, Purkinje cell layer, and granular layer. This finding is in agreement with the work of Ezi et al. [18], who also reported intact cerebellar cortex layers for the control group. Photomicrograph of the cerebellum in tramadol-treated rats revealed significant dose-dependent neurodegenerative changes. The most notable histopathological changes were observed in the Purkinje cells, which play a crucial role in motor coordination and cognitive functions. These cells exhibited signs of atrophy, loss of structural integrity, and disorganization, particularly in the high-dose group. These findings are consistent with previous studies, such as those by Cunha-Oliveira et al. [19], which reported that opioid exposure could lead to significant neuronal damage and degeneration, particularly in regions of the brain involved in motor control. The mild disruption of Purkinje cells observed in this study at high-dose (75 mg/kg of tramadol) underscores the vulnerability of the cerebellum to tramadol-induced neurotoxicity, potentially leading to impaired motor and cognitive functions.

Histology of the cerebrum

The histological analysis of the cerebral cortex in this study revealed varying structural changes among the different groups, particularly in the presence of vacuolations, necrotic nuclei and pyknosis, as well as the integrity of pyramidal cells.

In the control group (Group I), the normal histological architecture of the cerebral cortex with prominent pyramidal cells suggests that no neurodegenerative damage occurred, which is consistent with findings by various studies that identified healthy cerebral tissue by the presence of intact pyramidal neurons and minimal vacuolations [17]. In Group II, the mild pyknosis and vacuolations observed, along with necrotic nuclei, indicate the early stages of neuronal degeneration, which are consistent with previous studies that identified pyknosis (condensed, shrunken nuclei) and vacuolations as hallmarks of early neurodegeneration [20].

The results from groups III and IV, which show an increased presence of vacuolations and pyknosis compared to Group II, suggest a progression in neurodegenerative damage. This pattern correlates with observations made in research on chronic neurodegenerative diseases, where vacuolation and pyknotic changes become more pronounced with advancing neuronal damage [21]. These histological features have been previously reported in models of severe neurodegeneration [20].

The limitation of this study is majorly the experimental period. The study was conducted for three weeks, which may not capture the long-term effects of tramadol ingestion/abuse. Future research should consider a longer duration of administration.

## Conclusions

In conclusion, this study demonstrated that administration of tramadol induced mild aggression-like behavior in the experimental Wistar rats. Administration of increasing doses of tramadol has little effect on the antioxidant enzymes assayed. Mild histological changes in the form of vacuolation, cellular degenerations, necrotic nuclei, and pyknosis were seen in the cerebral cortex, while the cerebellar cortex was not affected histologically following administration of tramadol in adult male Wistar rats. Hence, tramadol at the present therapeutic doses had mild neurotoxic effects on adult male Wistar rats.
